# Risk of Swine Influenza Virus Spillover at the Human-Swine Interface – a Scoping Review

**DOI:** 10.3389/ijph.2025.1608380

**Published:** 2025-09-19

**Authors:** Sendhilkumar Muthappan, Rizwan Suliankatchi Abdulkader, Gulam Mohd, Jasmine Beryl Lydia, Janana Priya, Anusha Salvankar, Pujitha Mallina, Vineetha Varanasi, Manickam Ponnaiah, Subarna Roy, Manoj V. Murhekar

**Affiliations:** ^1^ ICMR-National Institute of Epidemiology, Chennai, Tamil Nadu, India; ^2^ ICMR-National Institute of Traditional Medicine, Belagavi, Karnataka, India

**Keywords:** swine, swine influenza virus, pandemic, influenza A virus, spillover

## Abstract

**Objectives:**

We conducted this scoping review to describe the factors that influence the risk of spillover of Swine Influenza Virus (SIV) at various human-swine interfaces.

**Methods:**

We used the PubMed and EMBASE databases to identify relevant articles published until February 2024. We included cross-sectional, case-control, cohort, randomized controlled trials (RCTs), and ecological studies. Two authors screened the titles, abstracts, and full texts. The extracted details were presented in tables and figures.

**Results:**

Among the 55 studies, the majority were conducted in the United States (n = 27) and published after 2015 (n = 30). Occupational risk factors were the most commonly reported (n = 14), followed by lack of biosecurity measures (n = 10). We classified the identified risk factors into two broad categories: (1) risk factors that influence the transmission of SIV among swine and from swine to human, and (2) risk factors associated with the type of human-swine interfaces.

**Conclusion:**

Vaccination, biosecurity measures, and surveillance systems at human-swine interfaces effectively reduce swine influenza transmission. These strategies can be tailored to specific risk factors in common interaction settings.

## Introduction

Swine influenza is a respiratory disease in pigs caused by type A influenza viruses, which regularly lead to outbreaks of influenza among the pig population. The most common subtypes of Swine Influenza Virus (SIV) currently circulating in pigs are H1N1, H1N2, and H3N2 [1]. SIVs pose a significant public health risk and economic burden due to their potential to spill over to humans and other animals, potentially leading to pandemics. They occur in an epizootic or enzootic form, and hold greater consequences from the viewpoint of both animal and public health [2]. Infected pigs with swine influenza usually have a morbidity rate up to 100% and low mortality; however, in naive pigs, the mortality rate might rise to 10%–15% [3].

The epidemiology of SIV varies both within and across countries due to factors such as pig density, climatic conditions, and farming practices [4]. Swine serves as a potential source of IAV infection in livestock workers and their subsequent transmissibility to households of such workers [5]. It has been well documented that SIV has the potential to develop into an influenza pandemic through human-to-human transmission. Swine influenza outbreaks are frequently noticed in North and South America, Europe, and Asia. Since 2005, the United States of America (USA) has faced sporadic human infection with SIV, which is attributed to the mixing and co-circulation of triple-reassortant H3N2 rooted with swine lineages that further generated H1N1 and H1N2 reassortant swine viruses [6, 7]. The first swine-origin influenza A (H1N1) virus with the potential to turn into a pandemic emerged in Mexico in 2009 [8].

Spillover transmission occurs when an animal pathogen infects a human. The combination of multiple factors, including pathogen exposure, disease dynamics in the reservoir host, and human factors that impact susceptibility to infections, determines the chance of spillover [9]. Industrialization, urbanization, and changing agricultural landscape have further increased the interfaces between wild/water birds and domesticated birds (poultry), as well as between wild/migratory birds and humans. The evolution of the 2009 H1N1 pandemic virus, which contained gene segments from European and North American swine lineages, underscores the urgent need to understand the risk of spillover and improved surveillance and preparedness [10–12]. In order to predict and prevent future outbreaks and pandemics, it is essential to understand the factors that increase the risk of spillover between swine and humans. Hence, we conducted this scoping review with the objective of mapping and describing the factors that increase the risk of spillover of swine influenza viruses at human-swine interfaces.

## Methods

We followed the Preferred Reporting Items for Systematic Reviews and Meta-Analyses – Scoping Reviews (PRISMA-ScR) guideline for this review.

### Eligibility Criteria

We included cross-sectional studies, case-control studies, cohort studies, randomized controlled trials (RCTs), and modelling studies that investigated the risk factors associated with SIV spillover at the human–swine interface. Articles were eligible if they were published up to February 2024. We excluded conference abstracts, editorials, opinion pieces, and articles not published in English.

### Search Strategy

We conducted a comprehensive literature search using PubMed and EMBASE to identify relevant articles. Key search terms included Influenza, Swine, Human, Zoonosis, Animal, Interfaces, Risk Factors, and Spillover. The retrieved articles were imported into Rayyan for duplicate removal and screening. We used two reviewers for title/abstract screening and full-text screening. Both reviewers received training in the use of Rayyan, and individual login credentials were created to ensure independent access and blinded screening. The detailed PubMed search strategy is provided in Supplementary Appendix S1.

### Study Selection

We followed a two-stage screening process. Two authors, JP and JL, independently assessed all titles and abstracts, following the removal of duplicates, to identify studies eligible for full-text screening. We included the studies that investigated the risk of spillover at human-swine interfaces. The same authors were involved in full-text screening, and all full-texts were re-assessed against the key inclusion criteria. Disagreements that surfaced during the full-text, title, and abstract screening were resolved by the third author.

### Data Extraction and Synthesis

We used a data extraction form to obtain pre-specified details from the included articles using Microsoft Excel. Three authors were involved in data extraction (JP, JL, AS). Consensus was sought between the three extracting authors in cases of conflict. We extracted details such as author, year of publication, country, study design, study sample, interface or study setting, virus details, risk factors, key findings, and risk factor category, and summarized the findings as frequencies.

## Results

### Screened and Included Studies

**FIGURE 1:**
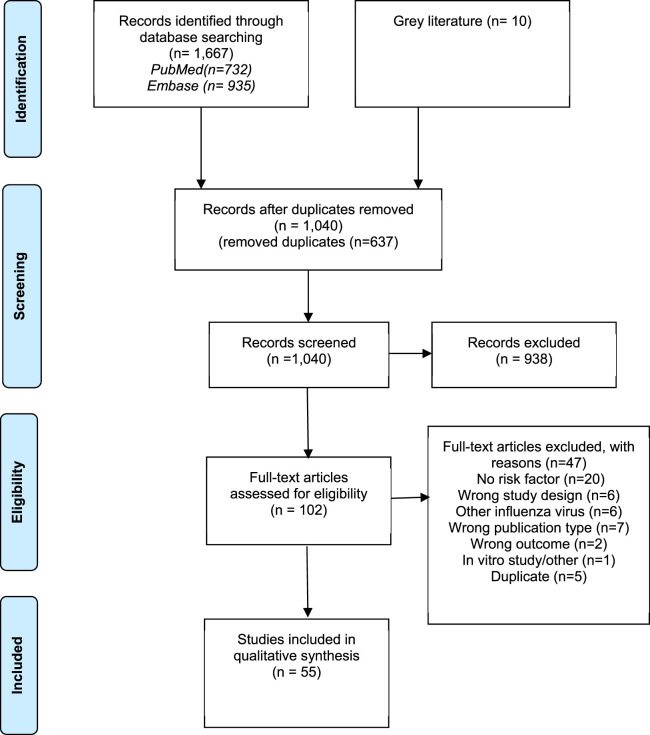
Flow diagram summarizing the study selection process (Global, 2024).

We included 1,667 articles from two databases and 10 from grey literature. We removed duplicates (n = 637) and included 1,040 articles for screening. During title and abstract screening, we excluded 938 articles and included 102 articles for full-text screening. Further, 47 articles were [No risk factor (n = 20); wrong study design (n = 6); other influenza virus (n = 6); wrong publication type (n = 7); wrong outcome (n = 2); *in-vitro* study (n = 1); duplicates (n = 5)] excluded based on the inclusion and exclusion criteria during the full-text screening. We included 55 articles for data extraction. The selection process is represented in the PRISMA flow diagram (Figure 1).

### Characteristics of Included Studies

**FIGURE 2:**
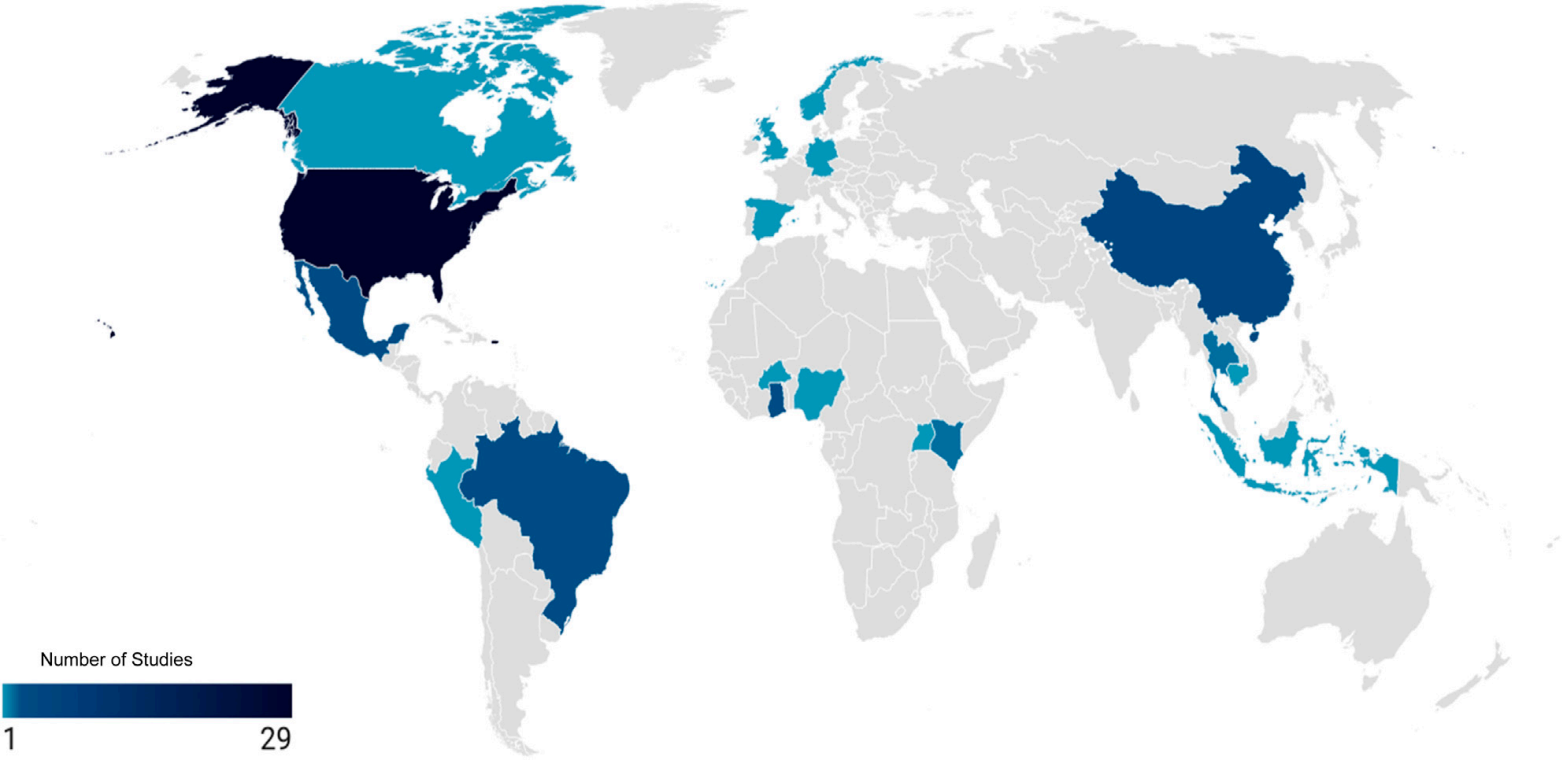
Global distribution of research studies on risk of spillover in swine-human interfaces (Global, 2024).

Most studies were from the USA (n = 27), followed by China (n = 6), Mexico (n = 4), and Brazil (n = 3) (Figure 2). Most of the studies were published after 2015 (n = 30). Most of the studies reported occupational risk factors (n = 14), followed by lack of biosecurity (n = 10) and environmental (n = 6). A large majority of the studies were cross-sectional studies (n = 28), followed by cohort studies (n = 3). More than one-fifth of studies were done on agricultural fairs (n = 13), followed by large-scale commercial farming (n = 12), and backyard farming (n = 8) (Supplementary Table 1). We classified the risk factors into two broad categories: (1) risk factors that influence the transmission of SIV among swine and from swine to human, and (2) risk factors associated with the type of human-swine interfaces. The first category includes factors such as pig attributes and rearing practices, the extensiveness of interfaces, occupational exposure, environmental conditions, host factors, and lack of biosecurity measures. The second category focuses on the specific settings where such interactions occur, which include backyard farming, mixed farming systems, large-scale or commercial pig farms, live animal markets and slaughterhouses or abattoirs, swine exhibitions and agricultural fairs, and veterinary hospitals, clinics, or research facilities.

### Pig Attributes and Rearing Practices

Several studies reported that age, sex, and rearing practices of pigs significantly influence susceptibility to SIV infection [13, 14, 45, 64]. Younger pigs were consistently reported to have higher odds of SIV positivity compared to older pigs [14, 45, 64]. For instance, piglets aged 1–10 days demonstrated significantly greater odds of infection [14], and SIV was most frequently isolated from weaned piglets aged 4–8 weeks [45]. In addition to age, sex was identified as a contributing factor; female pigs had higher seroprevalence than males (PR = 2.84) [13]. Other factors such as a high number of breeding sows (OR = 3.98) [23], free-ranging swine in contact with domestic ducks and wild birds [61], animals from outside sources, and the presence of crossbred pigs were similarly linked to elevated SIV risk and seroprevalence.

### Extensiveness of Interface

Several studies highlight that close and repeated human-swine contact in settings such as agricultural fairs, exhibitions, and backyard farming facilitates viral spillover. For instance, one study reported that even a small proportion of IAV-positive swine arriving at fairs could contribute to transmission dynamics [53], while another observed frequent human-swine contact in backyard systems as a potential risk factor [59]. The unique setting of agricultural fairs enables sustained close interactions with swine, thereby amplifying the potential for interspecies transmission [38, 39, 47, 51, 63, 65]. Duration of exposure also plays a crucial role. One study identified significant associations between hours spent with pigs (R^2^ = 0.90, P = 0.0018) and with pigs from different farms (R^2^ = 0.91, P = 0.0001) and the presence of IAV [60]. The risk of suspected zoonotic infection increased with intensity of swine contact, ranging from no exposure to visiting swine exhibits to direct physical contact [32]. Additionally, the scale of swine operations and the degree of trade connectivity were associated with heightened transmission risk [16, 31]. The attitude towards and awareness about recommended practices also play a key role [55]. However, not all studies reported definitive associations. For example, one study found concurrent IAV circulation among pigs, poultry, and humans on farms, but did not identify significant risk factors for human-to-swine transmission [50].

### Occupational Risk

Occupational exposure significantly contributes to the spillover of influenza viruses at the human-swine interface due to frequent, close, and often unprotected contact with pigs. Several studies reported that individuals working on swine farms had markedly higher odds of infection [19, 21, 35, 37, 42], particularly among farm residents [20], swine workers [18, 20, 22, 36, 52, 57, 67], contact among wild species and swine [44], and those employed in the pig industry [21, 66]. Farm workers were more likely to test positive at the end of the working day (OR = 1.98; 95% CI: 1.14–3.41) [24], and swine workers showed significantly higher seroprevalence of swine H3N2 (17.3% vs. 7.0%; adjusted OR = 3.4; 95% CI: 1.1–10.7) [28]. The veterinarian also has a high risk of transmission [19, 20]. One study reported that the cumulative incidence of acute respiratory illness was high among pig workers [34]. Specific occupational tasks such as walking the aisles (27%), handling pigs (21%), and handling contaminated equipment (21%) also increased the risk of SIV transmission [46].

### Environmental Factors

Environmental conditions such as temperature, humidity, seasonality, and airflow play a critical role in influencing the transmission dynamics of influenza viruses. One study reported a significant association between temperature and humidity with the presence of antibodies against H1N1 and H3N2 strains in pigs, suggesting that these factors impact viral circulation. This study reports that for every degree Celsius increase in average temperature, pigs had 2.26 times higher odds of having positive titres for the virus (p < 0.05, CI 1.22, 4.18) [29]. One study reported a significant association between temperature and humidity with the presence of antibodies against H1N1 and H3N2 strains in pigs, suggesting that these factors impact viral circulation. This study reports that for every degree Celsius increase in average temperature, pigs had 2.26 times higher odds of having positive titres for the virus (p < 0.05, CI 1.22, 4.18) [25]. Specific environmental conditions were linked to higher IAV positivity rates. Detection was increased at outdoor temperatures of 5.0 °C–13.9 °C (OR = 3.06; 95% CI: 1.04–8.98) and 14.0 °C–23.9 °C (OR = 3.44; 95% CI: 1.08–10.95). Additionally, IAV detection rates were elevated in summer (OR = 3.32; 95% CI: 1.16–9.50) and fall (OR = 4.12; 95% CI: 1.47–11.54) [14]. Other factors, such as the presence of wild birds and poultry, were also associated with a high risk of SIV [29]. Similarly, a study found that seasonal trends were also evident, with a smaller peak from May to July (24%). In contrast to this, it was also found that the highest infection risk was observed between January and March (accounting for 54% of estimated peaks). Although some level of infection risk was present throughout the year, infection trends were positively correlated with humidity and closely mirrored influenza patterns in domestic swine and human populations [26].

### Host Factors

Host factors in infectious diseases refer to the biological, physiological, and immunological characteristics of pigs or humans that influence susceptibility to infection, viral shedding, and transmission dynamics. Few studies have highlighted such factors in the context of swine influenza. For example, the age and vaccination status of veterinarians were significantly associated with seropositivity for swine-origin influenza viruses [27, 28]. Similarly, limiting the duration of swine exhibitions (e.g., ≤72 h) was shown to drastically reduce IAV prevalence in exhibition swine [41]. Additionally, the presence of influenza-like illness (ILI) among individuals with swine contact was linked to higher seroprevalence [28]. Human population density has also been identified as a contributing factor to the increased risk of infection [58]. Furthermore, ecological factors such as the presence of wild birds and domestic swine populations near human settlements were associated with elevated seropositivity rates [56] underscoring the complex interplay between host characteristics and environmental exposure in shaping disease risk.

### Lack of Biosecurity

Multiple studies have demonstrated that the absence of adequate biosecurity measures is a key driver of interspecies transmission. For instance, farms lacking biosecurity protocols, poor husbandry, and a lack of biosafety practices for workers exhibited a higher risk of infection, underscoring the urgent need for stronger preventive practices [17, 29, 33, 43, 48, 49, 54, 62]. One intervention study reported a 21% reduction in viral positivity when sow vaccination was combined with enhanced biosecurity, indicating that integrated approaches can effectively reduce IAV transmission and support the production of IAV-free piglets at weaning [30]. Furthermore, unrestricted access to farms has also been identified as a significant risk factor for disease spread [15]. In addition, the lack of structured mitigation strategies at agricultural fairs has been highlighted as a vulnerability, prompting calls for reinforced biosecurity to safeguard both animal and public health [40].

### Associated Risks by Type of Interface

#### Backyard Farming

In backyard farming settings, several factors have been associated with increased seroprevalence of IAV in swine. A study conducted in southern Brazil identified age, sex, the number of suckling pigs, and proximity to neighboring pig holdings as key contributors to IAV transmission risk, emphasizing the need for continued surveillance in such settings [13, 30]. Multiple studies have reported that poor biosecurity practices in backyard and small-scale farms significantly contribute to SIV transmission, underscoring the critical role of implementing proper farm hygiene and biosecurity measures to reduce the risk at the human-animal interface [54, 59, 60]. Moreover, a study highlighted that free-ranging swine practices are a major risk factor for human-to-swine spillover of influenza viruses [61].

#### Mixed Farming/Large-Scale or Commercial Farming

Occupational and environmental factors play a critical role in the transmission of influenza viruses in mixed, large-scale, and commercial farming systems. Key risk groups include swine farm workers [20, 22–24, 28] and veterinarians [20], whose direct and frequent contact with pigs increases the likelihood of zoonotic transmission. One study reported that occupational exposure to pigs was significantly associated with increased A(H1N1)pdm09 seropositivity (adjusted OR = 25.3; 95% CI: 1.4–536.3) [21]. Studies also indicate that low usage of personal protective equipment (PPE) contributes to higher SIV transmission risks in mixed farming systems [34]. On the other hand, studies show that sow vaccination and the implementation of enhanced biosecurity practices can effectively reduce IAV transmission among piglets and support the weaning of virus-free cohorts [30].

#### Live Animal Market/Slaughterhouses/Abattoirs

Continuous exposure of farm and abattoir workers to infected animals increases the risk of interspecies transmission of influenza viruses. Studies recommend the implementation of proper regulations and routine surveillance in live animal markets and slaughterhouses to mitigate this risk [36, 58]. Additionally, biosecurity measures have been shown to play a key role in reducing the threat of swine influenza in these settings [49].

#### Swine Exhibition/Agricultural Fair

Agricultural fairs present a significant risk for IAV transmission due to close and prolonged contact between large groups of humans and swine. Multiple studies have shown that attending swine exhibitions or agricultural fairs increases the risk of infection [38, 40, 65, 66]. One study reported higher infection risks associated with visiting swine exhibits (8%; RR 2.1; 95% CI: 0.2–53.4) and touching swine (16%; RR 4.4; 95% CI: 0.8–116.3). Infected pigs at fairs further elevate the risk of transmission [39, 53], and longer fair durations have also been linked to increased infection potential [41, 47]. These findings highlight the need for active surveillance, investigation of illness among attendees [32], and the implementation of strict biosecurity measures at such events [55].

#### Veterinary Hospitals/Clinics/Research Facilities

Veterinary healthcare settings expose workers to sick animals, including pigs and birds, increasing the risk of zoonotic disease transmission. A study of a pH1N1 outbreak at an Alberta research farm with 37 humans and 1,300 swine found that seven people developed ILI. It highlighted a significant association between seropositivity and those working in the swine nursery, indicating an occupational risk [42].

## Discussion

This scoping review identified key factors contributing to the risk of SIV spillover at human–swine interfaces, based on 55 eligible articles. Occupational exposure was a major risk, particularly among individuals directly handling pigs. Environmental conditions, such as temperature, humidity, and seasonal variations, also influenced viral circulation. Poor biosecurity, such as inadequate hygiene and unrestricted farm access, consistently emerged as a key risk factor, highlighting the need for targeted prevention strategies. We also found that different interface types, including backyard farms and agricultural fairs, contribute significantly to spillover risks.

Occupational and environmental exposures remain critical contributors to SIV transmission. Swine workers and individuals attending agricultural fairs face elevated risks due to prolonged and close contact with pigs [68]. In small, confined farm settings, such high-contact behaviors further elevate the risk of interspecies transmission [69]. Additionally, seasonal and ecological factors, including deforestation, play a role in altering animal-human interaction patterns, thus contributing to the emergence of zoonotic threats [32, 70–72]. Regular surveillance of farms and timely detection of IAV can help reduce spillover risk [73, 74]. Vaccination has proven effective in reducing the transmission of the virus at human–swine interfaces. Several studies have reported that influenza vaccination helps decrease susceptibility to infection, reduce transmission, and prevent future pandemics, thereby safeguarding public health [75–77]. A study found that pre-exhibition influenza vaccination of swine can reduce the public health risk posed by IAV at agricultural exhibitions [76]. Additionally, another study highlighted that vaccination not only limits virus replication in pigs but also protects public health by preventing the generation of novel reassortants with zoonotic and/or pandemic potential [75]. The findings underscore the critical role of occupational and environmental factors and targeted vaccination in mitigating the risk of SIV spillover at human-swine interfaces.

Our review identified the lack of biosecurity measures as a significant risk factor for the transmission of SIV [4, 78], a finding supported by several other studies. One study showed that enhanced biosecurity could reduce transmission risk by 50%, while further improvements led to a 79% reduction in infected pigs and a 74.8% decrease in infected humans [68]. Smaller-scale farms with outdoor access for pigs and weaker biosecurity were also reported to be at higher risk for disease outbreaks [79, 80]. A report from Kathmandu, Nepal, reported significant non-compliance with health codes in local slaughterhouses, elevating the risk of zoonotic diseases for butchers due to poor hygiene [81]. Effective hygiene practices, such as the use of personal protective equipment (PPE) and preventing sick employees from entering farms, have been shown to significantly reduce the risk of zoonotic spillover and the emergence of novel strains [78]. Additional studies emphasize the importance of PPE use, isolating sick animals, and enforcing quarantine and monitoring protocols, especially in contexts involving contact with other animal species [43, 67, 80]. A modeling study further suggested that shortening the duration of swine exhibitions, along with strengthened biosecurity measures, could reduce infection risks during agricultural fairs [75]. Implementing protective measures like bird-proof netting and livestock acclimatization can reduce disease transmission risks [44]. Improved biosecurity measures, including the use of proper PPE, adherence to health codes, hygiene practices, and strict quarantine enforcement, have proven effective in reducing the risk of SIV spillover at human–swine interfaces.

Interface types such as backyard farming, agricultural fairs, and large-scale farming pose significant risks for SIV spillover due to close and prolonged human–swine contact. Several studies recommend strengthening veterinary assistance and surveillance in backyard pig and poultry production to reduce transmission risks [82]. Enhancing virus detection capabilities in backyard swine and poultry systems has also been suggested as a priority for early identification and response [74]. Research from South America highlights the need for improved IAV surveillance in backyard settings, given the close interaction among domestic animals, wild birds, and humans in these environments [83]. A systematic review supports the regular monitoring of IAV in backyard swine populations to aid informed decision-making for sustainable farming and public health [84]. In the context of agricultural fairs, studies recommend mandatory influenza vaccination for pigs before exhibition [76] and shortening the duration of swine exhibitions to limit movement and potential transmission during fairs [65]. Surveillance, proper biosecurity measures, regular monitoring, and reducing the duration of swine exhibitions have been effective in limiting SIV transmission at human–swine interfaces.

### Limitations

This study has a few limitations. We included only articles published in English and retrieved data only from two databases. This paper discusses the risk factors associated with the SIV at the interface between human-swine. The design of the included studies varies, which limits the comparison between the studies. Furthermore, we were unable to rank the identified risk factors due to variations among studies in how they prioritized the risks.

### Conclusion

We identified several key factors contributing to the risk of SIV spillover from swine to humans. Frequent and close contact between humans and swine, inadequate biosecurity practices, and poor surveillance systems emerged as major risk factors. Additional contributing elements included specific pig-rearing practices, environmental conditions such as temperature and humidity, and occupational exposure among farm workers and veterinarians. Evidence suggests that implementing strict biosecurity measures, vaccinating both pigs and workers, and using PPE can significantly reduce transmission risk. These findings emphasize the need for comprehensive, multi-layered strategies to mitigate the spread of SIV and reduce the likelihood of future zoonotic outbreaks.
