# Supplementary material for: Risk of Swine Influenza Virus Spillover at the Human-Swine Interface – a Scoping Review
Source: Int J Public Health. 2025 Sep 19;70:1608380. doi: 10.3389/ijph.2025.1608380 (PMC12491070; doi:10.3389/ijph.2025.1608380)
Supplement: Supplementary file 1 [file Supplementaryfile1.docx]

**Annexure: Search Strategy in PubMed**

|  | | |
| --- | --- | --- |
|  |  |  |
| **Item** | **Search Strategy** | **No. of Articles** |
|  | **(PubMed)** |  |
| #1 Influenza | (Influenza zoono*[Title/Abstract]) OR (zoonotic Influenza[Title/Abstract]) OR (Swine influenza[Title/Abstract]) OR Influenza A[Title/Abstract] OR SIV[Title/Abstract] OR IAV[Title/Abstract] OR Influenza virus*[Title/Abstract] OR IAVs[Title/Abstract] | 72,316 |
| #2 Human | (Humans [Mesh]) OR (human*[Title/Abstract]) OR (Pig Farmer*[Title/Abstract]) OR (swine farmer*[Title/Abstract]) OR (pork farmer*[Title/Abstract]) OR (pig trader*[Title/Abstract]) OR (swine trader*[Title/Abstract]) OR (live market residents[Title/Abstract]) OR (pig handle*[Title/Abstract]) OR (Swine handle*[Title/Abstract]) OR (pig farm worker*[Title/Abstract]) OR (slaughter house worker*[Title/Abstract]) OR (swine worker*[Title/Abstract]) OR (pig worker*[Title/Abstract]) OR (swine farm worker*[ Title/Abstract]) OR (pig seller*[ Title/Abstract]) OR (swine seller*[ Title/Abstract]) OR (pork seller*[ Title/Abstract]) | 22,582,780 |
|  |  |  |
| #3 Swine | (Pig [Title/Abstract]) OR (Swine* [Mesh]) OR (Hog [Title/Abstract]) OR (Boar [Title/Abstract]) OR (Warthogs [Title/Abstract]) | 335,574 |
| #4 Spillover | (Spill over [Title/Abstract]) OR (Spillover [Title/Abstract]) OR (Transmission [Title/Abstract]) OR (Spread [Title/Abstract]) OR (Transmissibility [Title/Abstract]) OR (Spillage [Title/Abstract]) | 695,615 |
|  |  |  |
| #5 | #1AND #2 0R #3 AND #4  ((((Influenza zoono*[Title/Abstract]) OR (zoonotic Influenza[Title/Abstract]) OR (Swine influenza[Title/Abstract]) OR Influenza A[Title/Abstract] OR SIV[Title/Abstract] OR IAV[Title/Abstract] OR Influenza virus*[Title/Abstract] OR IAVs[Title/Abstract]) AND ((Humans [Mesh]) OR (human*[Title/Abstract]) OR (Pig Farmer*[Title/Abstract]) OR (swine farmer*[Title/Abstract]) OR (pork farmer*[Title/Abstract]) OR (pig trader*[Title/Abstract]) OR (swine trader*[Title/Abstract]) OR (live market residents[Title/Abstract]) OR (pig handle*[Title/Abstract]) OR (Swine handle*[Title/Abstract]) OR (pig farm worker*[Title/Abstract]) OR (slaughter house worker*[Title/Abstract]) OR (swine worker*[Title/Abstract]) OR (pig worker*[Title/Abstract]) OR (swine farm worker*[ Title/Abstract]) OR (pig seller*[ Title/Abstract]) OR (swine seller*[ Title/Abstract]) OR (pork seller*[ Title/Abstract]))) AND ((Pig [Title/Abstract]) OR (Swine* [Mesh]) OR (Hog [Title/Abstract]) OR (Boar [Title/Abstract]) OR (Warthogs [Title/Abstract]))) AND ((Spill over [Title/Abstract]) OR (Spillover [Title/Abstract]) OR (Transmission [Title/Abstract]) OR (Spread [Title/Abstract]) OR (Transmissibility [Title/Abstract]) OR (Spillage [Title/Abstract]) | 732 |
