# Supplementary material for: Risk of Swine Influenza Virus Spillover at the Human-Swine Interface – a Scoping Review
Source: Int J Public Health. 2025 Sep 19;70:1608380. doi: 10.3389/ijph.2025.1608380 (PMC12491070; doi:10.3389/ijph.2025.1608380)
Supplement: Supplementary file 2 [file Table1.docx]

**Table 1: Summary of articles included in the scoping review (Global, 2024)**

| **S.No** | **Author and Year** | **Country** | **Factors Investigated** | **Study design** | **Sample size** | **Interface/study setting** | **Virus** | **Spillover type** | **Subjects [Pig/Human /Environmental]** | **Key Finding.** | **Risk Factor Category** |
| --- | --- | --- | --- | --- | --- | --- | --- | --- | --- | --- | --- |
| 1 | Carine K. Souza et at., 2018.  [13] | Brazil | Factors associated with IAV seroprevalence in backyard pigs. | Cross-sectional study | 1,667 pig sera samples | Backyard farming | H1N1pdm09, H1N1, H1N2 and H3N2 | Swine to Swine | Pig | Age, sex, the number of suckling pigs, and nearby pig farms as key factors affecting IAV seroprevalence. | pig attributes |
| 2 | Benjamin D. Anderson et al.,2018. [14] | China | Factors affecting IAV shedding, IAV positivity on environmental surfaces and bio-aerosol IAV positivity | Prospective study | 396, each of environmental swab, water, bioaerosol, and fecal/slurry specimens, and 3,300 pig oral secretion specimens | Pig farms | IAV | Swine to Swine | Pig, human, environmental | Presence of IAV in pigs (7.1%) and their environment (up to 11.6%), which highlights the zoonotic spillover risk associated with environmental contamination and inadequate biosecurity. | Occupational; Environmental |
| 3 | Simon-Grife´ et al.,2010. [66] | Spain | Seroprevalence and risk factors of swine influenza | Cross-sectional study | 2,151 pigs from 98 farms | Pig farms | H1N1, H1N2, H3N2 | Swine to Swine | Pig | High seroprevalence of SIV viruses (H1N1, H1N2, H3N2) was identified in the majority of pigs (75.4%), with mixed infections found on 89.8% of farms. | Lack of biosecurity |
| 4 | Paul M. Lantos et al.,2016. [29] | USA | Factors amplifying the transmission of the influenza virus (H1N1) to humans | Survey | 7,41,276 humans | Swine farms | Pandemic 2009 H1N1 | Swine to human | Pig and Human | People living near modern swine production facilities had increased odds of ILI | Environmental; occupational |
| 5 | Nurhayati et al.,2020. [58] | Indonesia | Risk factors for Swine Influenza (H1N1) seropositivity at the farm level | Cross-sectional study | 649 serum samples (175 pig farms) | Large-scale farms | SIV | Swine to human | Pig | Herd-level SIV seropositivity was 26%, with increased risk linked to other animals, nearby poultry, and pig collectors. | extensiveness of the interface, Environmental |
| 6 | Gregory C. Gray et al.,2006. [39] | USA | Direct occupational exposure and indirect exposure to close family members | Prospective study | 707 AHS swine-exposed, 80 AHS non-swine-exposed. 79 University of Iowa controls | Mixed farming | SwH1N1 SwH1N2 | Swine to human | Human | Workers exposed to swine (OR=54.9) and their non-exposed spouses (OR=28.2) showed elevated antibody levels to swine H1N1, indicating a higher risk of zoonotic infection. | Occupational |
| 7 | Myers KP et al.,2006. [33] | USA | Occupational exposures | Cross-sectional study | 352 Human (111 farmers, 97 meat processing workers, 65 veterinarians, and 79 control subjects) | Mixed farming | SwH1N1 and SwHIN2 | Swine to human | Pig and Human | Occupational groups had significantly higher odds of prior swine IAV infection, with the highest odds among farmers (OR=35.3 for H1N1). | Occupational |
| 8 | Han Zhou et al.,2014. [38] | China | Occupational exposures | Sero-epidemiological study | 546 humans and 1,180 pigs | Large Scale/Commercial Farming | Avian like A(H1N1) SIV | Swine to human | Pig and Human | 11.17% of swine farm residents in southern China tested positive for avian-like A(H1N1) SIV, which is significantly higher than that of urban controls (P = 0.031). | Occupational |
| 9 | Ellen Fragaszy et al., 2016 [37] | UK | Occupational exposure, seropositivity in pigs/humans | Cross-sectional study | 17 farms | Commercial pig farms | A(H1N1)pdm09 | Swine to human | Human | Occupational exposure to pigs significantly increased A(H1N1) pdm09 seropositivity (aOR = 25.3; 95% CI: 1.4–536.3). | Occupational |
| 10 | Carl Andreas Grøntvedt et al.,2013. [44] | Norway | Factors affecting the introduction of IAV infection to pig farms | Cross-sectional study | 118 pig herds | Large Scale/Commercial Farming | Influenza A(H1N1) pdm09 virus | Human to Swine | Pig and Human | Having farm staff with ILI (OR=4.15) and a larger herd size (OR=1.01) were identified as significant risk factors for pig infection. | Occupational |
| 11 | G. Lo´ pez-Robles et al., 2011. [17] | Mexico | Occupational exposure and factors affecting zoonotic transmission of SIV | Cross-sectional study | 125 (62 swine workers, 63 controls) | Large Scale/Commercial Farming | SwH3N2 and SwH1N1 | Swine to human | Human | Farm workers showed higher seroprevalence of swine influenza subtypes, especially H3N2, linked to swine exposure and farm location. Influenza vaccination also demonstrated a protective effect. | Pig attributes and Occupational |
| 12 | Lopez-Moreno .G. et.al., 2022. [47] | USA | The risk of IAV detection in swine farmworkers | Cross-sectional study | 7 swine breeding herds, herd inventories ranged between 1,500–4,000 breeding females, 2,000–6,000 suckling piglets, and 64 swine workers from 7 swine farms, 1,814 Nasal Swabs from farm workers | Large Scale/Commercial Farming | H1N1 | Swine to human | Pig and Human | 57% of swine farmworkers tested positive for IAV at least once; 41% tested positive after work. Genetic evidence of both human-origin and swine-origin IAV was detected in the workers. | Occupational; |
| 13 | Ferreira et al.,2017. [51] | USA | Micro-climatic factors affecting exposure to the Influenza viruses | Cross-sectional study | 450 pigs | Large Scale/Commercial Farming | SwH1N1_P, SwH3N2_D | Swine to Swine | Pig | There was a significant association between environmental temperature and humidity with the presence of antibodies to H1N1 and H3N2 strains in pigs. | Environmental |
| 14 | Kim M. Pepin et al., 2019. [53] | USA | Dynamics of IAV; Identification of risk factors associated with IAV infection in wild pigs;meteorological conditions | Surveillance study | Sero-surveillance data from wild pigs in 15 | Backyard farming | IAV | Swine to Swine | Pig | The risk of influenza A virus (IAV) infection in wild pigs peaked between January and March, and was associated with higher humidity levels and concurrent IAV activity in domestic swine and human populations. | Environmental |
| 15 | Montan˜ez et al., 2017. [54] | Mexico | Occupational exposure among veterinarians | Cross-sectional study | 81 serum samples of veterinarians | Large Scale/Commercial Farming | SwH1N1, SwH3N2 | Swine to human | Human | Age and vaccination status were associated with seroprevalence | Host factor |
| 16 | Mengmeng Ma et al.,2015. [48] | China | Factors affecting SIV infection in humans | Cross-sectional study | 318 (203 swine workers and 115 control subjects) | Large Scale/Commercial Farming | Swine H3N2 virus | Swine to human | Human | Swine workers demonstrated higher seropositivity to swine H3N2 virus. Significant risk factors included younger age, recent history of respiratory illness, and seropositivity to seasonal H3N2 influenza. | Host factors: occupational |
| 17 | Li Y et al.,2019  [52] | China | Risk factors for farmer-reported SI: wild bird access, poultry presence, lack of worker biosecurity | Cross-sectional study | 153 owners/managers of piggeries | Swine farming | Swine influenza | Swine to human | Human | Wild bird access, poultry on pig farms, and poor biosecurity for workers increased the risk, highlighting the need for better biosecurity and local husbandry awareness. | Lack of Biosecurity and environmental |
| 18 | Ustavo Lopez-Moreno et al., 2022G. [65] | USA | Impact of internal biosecurity practices | A controlled trial with treatment and control groups. | 360 litters | Large Scale/Commercial Farming | IAV | Swine to Swine | Pig | Combined sow vaccination and enhanced biosecurity reduced IAV detection, enabling the weaning of IAV-negative pigs | Lack of Bio-security |
| 19 | Mateus-Anzola.J. et. al., 2019. [30] | Mexico | Pig trade network in the transmission of influenza | Modelling study | 260 backyard farms as nodes | wildlife/livestock interface, backyard | Influenza A virus | NA | NA | High connectivity through informal pig trade leads to rapid and widespread influenza outbreaks, infecting half of the pig population within five days and significantly increasing the number of affected farms. | Backyard farming: modelling study |
| 20 | Karen K. Wong et al., 2012.  [28] | USA | Exposure to pigs during an agricultural fair | Retrospective Cohort Study | 127 humans attended the fair | Agricultural fair | Influenza A(H3N2) v | Swine to human | Human | The risk of infection increased proportionally with the level of swine exposure. | Extensiveness of interface |
| 21 | Matt W. Allerson et al., 2013. [60] | USA | Factors associated with the Indirect transmission of IAV | Experimental study | 35 pigs | Animal Isolation Facility | IAV | NA | Pig | Fomites can transmit IAV between pig groups; infection occurred in sentinel pigs despite biosecurity measures, highlighting the indirect transmission risk. | Bio-security |
| 22 | Eric Mogaka Osoro et al., 2019. [49] | Kenya | Association between occupational exposure to pig | Cohort study | 625 participants [172 pig workers, 453 non-pig workers] | Small-scale farming | IAV | Swine to human | Pig and Human | Moderate seropositivity for influenza A in pigs indicates virus circulation; however, pig workers experienced lower acute respiratory infection (ARI) incidence, although interspecies transmission potential remains. | Occupational |
| 23 | Paccha et al.,2016. [35] | USA | Occupational exposure to swine workers | Modelling study | 2,000 pigs (6 to 12 weeks old) | Large Scale/Commercial Farming | IAV | Swine to human | Human | Swine workers are at risk of IAV infection through both airborne and direct contact transmission during outbreaks. While N95 respirators reduce this risk, they do not provide complete protection. | Modelling study: Occupational |
| 24 | Ariane Ribeiro Amorim et al.,2013. [40] | Brazil | Risk of human/animal infection from asymptomatic SIV-infected pigs | Cross-sectional study | 330 piglets | Abattoir | IAV | Swine to human | Pig | 9% of asymptomatic piglets tested positive for IAV in an abattoir setting | Occupational risk: Live animal market; slaughterhouses/abattoirs |
| 25 | Adeola .O.A. et al., 2019. [34] | Ghana | Socio-environmental factors contribute to the occurrence and distribution of influenza-like illness (ILI) among swine industry workers | Cross-sectional study | 87 consenting pig handlers | Commercial and mixed farming | Influenza A(H1N1)pdm09 viruses | Swine to human | Pig and Human | Influenza A(H1N1)pdm09 was detected in pig handlers, with viral sequences showing genetic similarity to strains circulating in pigs, suggesting the potential for bidirectional transmission between humans and swine. | Environmental factor |
| 26 | Dillon S. McBride et al.,2021. [26] | USA | Risk factors associated with IAV detection in swine exhibitions | Cross-sectional study | 17,009 pigs | Agricultural fair | IAV | Swine to human | Pig and Human | IAV was detected in 13.9% of pigs at jackpot shows, indicating that these shows spread infections to county fairs. | Lack of biosecurity |
| 27 | Andrew S. Bowman et al., 2014.  [24] | USA | Identify fair-level risk factors | Cross-sectional study | 40 pigs | Agricultural fair | NA | Swine to swine/human | Pig | Larger swine show size and presence of breeding swine increased the odds of IAV in pigs at fairs | Extensiveness of interface |
| 28 | Lauterbach et al., 2018. [67] | USA | Potential of IAVs to contaminate the air and portable animal-care items | Surveillance study | 59 air samples & 400 surface samples | Agricultural fair | IAV | Swine to human | Environmental | IAV was detected in air and on surfaces in swine barns at fairs, indicating potential for environmental transmission | Agricultural fair |
| 29 | McBride .D.S.et.al., 2022. [55] | USA | The effectiveness of shortening swine exhibitions to <72 hours to reduce IAV risk. | Longitudinal study | 39,768 nasal wipes from 6,768 pigs; | Agricultural fair | H1N1, H3N2, H3N2 | Swine to human | Pig | Shortening swine exhibition duration to <72 hours significantly reduces IAV prevalence among pigs at county fairs | Extensiveness of interface |
| 30 | Sarah E. Forgie et al., 2011. [36] | Canada | Investigation of a pH1N1 outbreak on a swine research farm | Observational study | 37 humans and 1300 swine | Veterinary Hospitals | Pandemic influenza A (pH1N1) | Swine to human | Pig and Human | Human-to-swine transmission of pH1N1 occurred with mild illness in both species; working in the swine nursery was associated with seropositivity in humans | Research facilities |
| 31 | Beaudoin, A et al., 2012. [61] | USA | Influenza infections and biosecurity measures | Survey | 45 veterinarians, 426 influenza-positive specimens and 153 swine farms | Large Scale/Commercial Farming | H1N1, H3N2, H1N2 | Swine to human | Pig and Human | Influenza A subtypes H1N1, H3N2, and H1N2 were common in Minnesota swine herds; inconsistent use of protective equipment and low employee vaccination increases zoonotic transmission risk | Lack of biosecurity |
| 32 | Ana PaulaSerafini Poeta Silvaa et al.,2019. [45] | Brazil | Biosecurity measures | Cross-sectional study | 40 herds and 35,000 sows | Large Scale/Commercial Farming | H1N1pdm09, H1N2, H3N2 | Swine to Swine | Pig | Implementing biosecurity measures, such as bird-proof netting and gilt acclimatization units, was linked to a decrease in IAV seroprevalence, while using external replacement stock was associated with a higher risk of infection. | Lack of biosecurity; pig attributes and rearing practices |
| 33 | Takemae et al., 2011. [15] | Thailand | Factors associated with IAV seroprevalence in backyard pigs | Serological surveillance | 731 nasal swabs; 641 serum samples | Large Scale/Commercial Farming | SIV | Swine to Swine | Pig | Piglets ≤8 weeks are the main source of SIV isolation; farm-to-farm pig introductions facilitate virus transmission in Thai farms | Pig attributes and rearing practices; Lack of biosecurity |
| 34 | Paccha B. et.al., 2016. [50] | USA | Behavior and precautions | Survey | 6 farms (2,400 to 12,000 pigs on site) | Large Scale/Commercial Farming | IAV | Swine to human | Pig and Human | Workers often wore boots and gloves, but N95 respirators were rarely used, and hand hygiene during close contact was insufficient. This highlights the need for better infection control practices. | Occupational |
| 35 | Karen K. Wong et al., 2013. [25] | USA | Transmissibility of H3N2v virus from Swine to humans. Burden of H3N2v infections among fair attendees | Cohort study | Cohort of 100 agricultural club members. Fair attendees with swine contact (estimated at 14,910 people). | Agricultural fair | Influenza A(H3N2) variant (H3N2v) virus | Swine to human | Human | The estimated probability of swine-to-human H3N2v influenza transmission increases with the duration of swine contact at agricultural fairs. | Lack of biosecurity |
| 36 | Matilda Ayim-Akonor et al., 2020. [64] | Ghana | Presence of IAVs in Swine and farmers, viral subtypes, serological prevalence, human-animal interface practices, and biosafety practices of farmers. | Cross-sectional study | 1,200 swine samples and 99 farmer samples,150 farmers | Backyard farming | IAVs, subtypes H1N1pdm09 and H3N2 | Swine to human | Pig and Human | Evidence of H1N1pdm09 in both pigs and farmers in Ghana suggests unidirectional human-to-swine transmission. | Lack of biosecurity |
| 37 | Yin Li et al.,2020.  [59] | China | Awareness and practices around swine influenza, use of PPE, beliefs about zoonotic transmission, vaccination, illness behaviour | Cross-sectional study | 153 pig farmers, 21 pig traders, and 16 pig trade workers | local pig farms, live pig markets, and slaughterhouses | swine influenza (SI) | Swine to human | Pig and Environmental | Low awareness of SI zoonotic risk linked to unsafe behaviours; belief SI causes pig death and working while sick increased odds of poor protective practices; PPE use was low among those unaware of zoonotic potential. | Lack of biosecurity |
| 38 | Müller-Theissen.M.L.et.al., 2022. [32] | Mexico | To identify community-level exposure and circulation of IAV | Longitudinal study | 90 different pig herds; 137 households | Backyard farming | H3N2 H1 pdm09 | Swine to human | Pig and Environmental | Concurrent IAV circulation in backyard pigs and ducks in wetland settings suggested potential human-to-swine spillover. | Extensiveness of interface |
| 39 | Nelson .M.I.et.al., 2020. [21] | USA | The dynamics of IAV transmission through exhibition swine | Cross-sectional study | 5,704 from Swine at 113 exhibitions in 9 USA. states | Agricultural fair | H1N2 (H1-2) H1δ-2 | Swine to swine/human | Pig, human, environmental | Early-season swine shows act as centers for IAV introduction and spread, increasing the risk of zoonotic transmission. | Extensiveness of interface |
| 40 | Tialla. .D.et.al., 2020. [43] | Burkina Faso | Investigation of the past exposure of pigs to IAV; practices of people with occupational contact with pigs and their knowledge of influenza | Cross-sectional study | 41 pig farms; Nasopharyngeal swabs from 358 humans and from 600 pigs | Backyard / Extensive and Semi-intensive Pig Farming | H1N1 | Swine to human | Pig and Human | Serological evidence in pigs shows exposure to pandemic H1N1/2009, likely from humans due to poor protective practices. | Occupational |
| 41 | Bliss. N. et.al., 2016. [19] | USA | IAV prevalence among exhibition swine entering fairs | Cross-sectional study | Snout wipes were collected from 3,547 swine | Agricultural fair | H1N1 H3N2 | Swine to human | Pig, human, environmental | 5.3% of swine arrived at fairs already infected with IAV; potential fomite transmission observed via shared contact surfaces. | Lack of biosecurity; swine exhibition |
| 42 | Rabinowitz. P.et.al., 2013. [62] | USA | To determine zoonotic influenza awareness and precautions among swine workers | Convenience survey | Subjects from Connecticut and Massachusetts; N = 14 and Nebraska -N = 39 | Large and small commercial farms | NA | NA | Human | Swine workers exhibited minimal concern regarding zoonotic influenza, showed inadequate adherence to PPE, and neglected national guidelines. | lack of biosecurity |
| 43 | Jacqueline M. Nolting et al., 2019. [31] | USA | Swine exhibitor perceptions and adoption of swine production practices | Cross-sectional study | 155 participants | Agricultural fair | Variant IAV | Swine to human | Pig and Human | Despite awareness of IAV risk, swine exhibitors often resist adopting recommended behaviours, including avoiding food in barns. | Lack of biosecurity; |
| 44 | Brigitte E. Martin et al., 2017. [57] | USA | seroprevalence and antigenic characterisation of IAV | Cross-sectional study | 8,239 serum samples from feral Swine | Mixed farming | IAVs, including both avian and swine strains | Feral Swine, wild birds, and domestic Swine (bi-directional) | Pig and Environmental | Feral swine have been exposed to both avian and swine IAVs, suggesting a risk of reassortment and potential spillover. | Host factor |
| 45 | Fatimah S. Dawood et al., 2011.  [42] | USA | Exposure to event pigs; case exposure; Seropositivity among exposed individuals | Cohort study | 42 serum samples, 17 seropositive individuals, 9 students, 8 index cases, 10 persons without exposures | Agricultural fair | Triple-reassortant SIV | Swine to human | Pig and Human | A pre-pandemic outbreak of triple-reassortant swine influenza occurred among students exposed to pigs at a livestock event, with no evidence of human-to-human transmission | Viral strain; occupational risk; host factor; extensiveness of interface |
| 46 | P. Netrabukkana et al., 2014. [56] | Cambodia | Association between seroprevalence of human influenza viruses in pigs and population density | Retrospective Cohort Study | 1147 serum samples from pigs | National Veterinary Research Institute (NaVRI); Institut Pasteur in Cambodia (IPC), and Slaughterhouses | Seasonal human H1N1 and H3N2 influenza viruses | Human to Swine | Pig and Human | Seroprevalence of human H3N2 influenza in pigs in Cambodia was positively associated with human population density. | Host factor; |
| 47 | Tinoco et al., 2016. [20] | Peru | Frequency of pH1N1 transmission between humans and swine on backyard farms | Cross-sectional study | 1,303 backyard swine | Backyard farming | Influenza | Human to Swine | Pig and Human | Human-to-swine transmission of pH1N1 occurred on backyard farms during the pandemic, confirmed by serology and viral isolation. | Extensiveness of interface |
| 48 | O. A. Adeola et al., 2017. [27] | Nigeria | Presence and spatial patterns of this human pandemic virus and identify associated risk factors. | Cross-sectional study | Pigs | Backyard farming | Influenza A(H1N1) pdm09 | Human to Swine | Pig and Human | Human-origin pH1N1 viruses were detected in Nigerian pigs, likely linked to extended contact with pigs and inadequate biosecurity. | Extensiveness of interface |
| 49 | Larison et al., 2014. [18] | USA | Swine Husbandry Practices. | Cross-sectional study | Pigs | Backyard farming | pH1N1 influenza | swine and other animal species. | Pig | Free-ranging pigs that frequently interacted with domestic ducks, wild birds, and humans faced a higher risk of pH1N1 spillover. | Pig attributes and rearing practice; |
| 50 | P. Netrabukkana et al., 2013. [63] | Thailand | Pig-Human contacts, bio-security Practices, | Cross-sectional study | 98 farmers and 5 traders | Backyard farming | IAV | Swine to human | Pig and Human | Extensive human-pig contact, commingling with poultry, and poor biosecurity in Thai smallholder farms increase risk of influenza A virus spread. | Pig attributes and rearing practice |
| 51 | Andrew S. Bowman et al., 2017. [22] | USA | Exhibition swine management practices, presence of human-like H3N2 IAV | Surveillance study | 161 pigs across the 7 fairs | Agricultural fair | IAV | Swine to human | Pig and Human | Influenza A(H3N2) virus transmission from exhibition swine to humans occurred at agricultural fairs, linked to reassortant virus strains | Extensiveness of interface |
| 52 | Michel Dione et al., 2018. [16] | Uganda | seroprevalence and identified the risk factors | Surveillance study | 522 clinically healthy pigs | Small-scale farming | IAV | Swine to Swine | Pig | High prevalence of bacterial and viral pathogens, including Influenza A, in smallholder pigs is linked to poor biosecurity practices. | Pig attributes and rearing practice |
| 53 | Andrew S. Bowman et al., 2012. [23] | USA | Subclinical infections, presence of IAV, genetic characteristics of Isolates | Surveillance study | 1,073 pigs | Agricultural fair | IAV | Swine to swine/human | Pig and Human | Subclinical Influenza A infections in pigs at agricultural fairs pose risk of undetected zoonotic transmission. | Extensiveness of interface |
| 54 | Rebekah S. Schicke et al., 2016 [46] | USA | Human exposure to infected swine, reassortant virus characteristics, and swine-human transmission | Epidemiological study | 372 H3N2v virus infections | Agricultural fair | Influenza A (H3N2v) | Swine to human | pig and human | 18 H3N2v cases with either direct or indirect swine contact. Of these, 16 infections were caused by a reassortant virus that contains a human-origin HA gene. Additionally, there has been no transmission from person to person. | Environmental |
| 55 | Jie Wu et al., 2015. [41] | China | Level of antibodies against CS H1N1 and H1N1pdm09 viruses. | Sero-epidemiological study | 712 participants | Occupational contact with Swine | A pandemic IAV; A CS H1N1 virus | Swine to human | Pig and Human | Swine workers had significantly higher antibodies against classical swine H1N1, but not 2009 pandemic H1N1. | Occupational |
