# Supplementary material for: Risk of Swine Influenza Virus Spillover at the Human-Swine Interface – a Scoping Review
Source: Int J Public Health. 2025 Sep 19;70:1608380. doi: 10.3389/ijph.2025.1608380 (PMC12491070; doi:10.3389/ijph.2025.1608380)
Supplement: Supplementary file 3 [file DataSheet1.pdf]

List of excluded articles

S. no	Article title	Reason
1	Bidirectional Human-Swine Transmission of Seasonal Influenza A(H1N1)pdm09 Virus in Pig Herd, France, 2018	No risk factor
2	Evolution of influenza A viruses in exhibition swine and transmission to humans, 2013-2015	Wrong outcome
3	Human Seasonal Influenza Viruses in Swine Workers in Lagos, Nigeria: Consequences for Animal and Public Health	No risk factor
4	Serologic evidence of human influenza virus infections in swine populations, Cambodia	No risk factor
5	Awareness and practices regarding zoonotic influenza prevention in Romanian swine workers	Wrong outcome
6	Evidence for Cross-species Influenza A Virus Transmission Within Swine Farms, China: A One Health, Prospective Cohort Study.	No risk factor
7	Airborne Influenza A Is Detected in the Personal Breathing Zone of Swine Veterinarians.	No risk factor
8	Vaccines That Reduce Viral Shedding Do Not Prevent Transmission of H1N1 Pandemic 2009 Swine Influenza A Virus Infection to Unvaccinated Pigs.	No risk factor
9	Spatial dynamics of human-origin H1 influenza A virus in North American Swine.	No risk factor
10	A stochastic compartmental model to simulate intra- and inter-species influenza transmission in an indoor swine farm.	Wrong study design
11	Molecular epidemiology of influenza A(H1N1)pdm09 virus among humans and Swine, Sri Lanka.	No risk factor
12	Transmission of swine influenza virus by lungworm migration.	In vitro

13	A serologic survey of pathogens in wild boar (sus scrofa) in sweden	No risk factor
14	Risk for interspecies transmission of zoonotic pathogens during poultry processing and pork production in Peru: A qualitative study.	Wrong study design
15	Study of influenza A virus in wild boars living in a major duck wintering site.	Wrong study design
16	Serologic survey of swine workers for exposure to H2N3 swine influenza A	No risk factor
17	Influx of Backyard Farming with Limited Biosecurity Due to the COVID-19 Pandemic Carries an Increased Risk of Zoonotic Spillover in Cambodia.	Study describes about other influenza virus
18	Evaluation of early single dose vaccination on swine influenza A virus transmission in piglets: From experimental data to mechanistic modelling.	Wrong study design
19	Pandemic influenza planning: shouldn't swine and poultry workers be included?	Wrong publication type
20	Do animal exhibitors support and follow recommendations to prevent transmission of variant influenza at agricultural fairs? A survey of animal exhibitor households after a variant influenza virus outbreak in Michigan.	No risk factor
21	Pig producers' perceptions of the Influenza Pandemic H1N1/09 outbreak and its effect on their biosecurity practices in Australia.	No risk factor
22	Pathogen exposure in feral swine populations geographically associated with high densities of transitional swine premises and commercial swine production.	No risk factor
23	Experimental infection of weanling pigs with A-swine influenza virus. I. Epidemiology and serological response.	No risk factor
24	Evidence of cross-reactive immunity to 2009 pandemic influenza A virus in workers seropositive to swine H1N1 influenza viruses circulating in Italy.	No risk factor
25	Multiple contributory factors to the age distribution of disease cases: a modeling study in the context of influenza A(H3N2v).	Wrong study design

26	Imported pigs may have introduced the first classical swine influenza viruses into Mainland China.	No risk factor
27	Influenza infection in humans and pigs in southeastern China.	No risk factor
28	Retrospective serological survey on selected viral pathogens in wild boar populations in Germany.	Study describes about other influenza virus
29	Experiences after Twenty Months with Pandemic Influenza A (H1N1) 2009 Infection in the Naïve Norwegian Pig Population.	No risk factor
30	Interspecies interactions and potential Influenza A virus risk in small swine farms in Peru.	No risk factor
31	Introduction, Evolution, and Dissemination of Influenza A Viruses in Exhibition Swine in the United States during 2009 to 2013.	No risk factor
32	Environmental surfaces used in entry-day corralling likely contribute to the spread of influenza A virus in Swine at agricultural fairs.	Wrong publication type
33	Evidence for subclinical avian influenza virus infections among rural Thai villagers.	Study describes about other influenza virus
34	Swine Backyard Production Systems in Central Chile: Characterizing Farm Structure, Animal Management, and Production Value Chain	Wrong population
35	Influenza A viruses are likely highly prevalent in South African swine farms	Wrong population
36	Using Environmental Sampling Techniques to Conduct Influenza A Virus Surveillance in Poultry and Waterfowl at Ohio Agricultural Exhibitions	Wrong population
37	Expansion of genetic diversity and interspecies transmission dynamics of swine influenza viruses in China	Wrong publication type
38	Investigation of the emergence of Avian and Swine Influenza among respiratory patients in Assiut University Hospital, Egypt	Study describes about other influenza virus
39	Influenza infections in live pig market, Nigeria	Wrong publication type
40	Risk of influenza A transmission at the pig-human interface in small pig farms in rural Thailand	Wrong study design
41	Serological evidence of pig-to-human influenza virus transmission on Thai swine farms	Study describes about other influenza virus

42	Swine influenza A at Fort Dix, New Jersey (January-February 1976). IV. Summary and speculation	Study describes about other influenza virus
43	Exposure of domestic Swine to influenza A viruses in Ghana suggests unidirectional, reverse zoonotic transmission at the human–animal interface	Duplicate
44	Characterization of influenza a outbreaks in Minnesota swine herds and measures taken to reduce the risk of zoonotic transmission.	Duplicate
45	Exploration of risk factors contributing to the presence of influenza A virus in Swine at agricultural fairs	Duplicate
46	Exposure of domestic Swine to influenza A viruses in Ghana suggests unidirectional, reverse zoonotic transmission at the human–animal interface	Duplicate
47	Exposure of domestic Swine to influenza A viruses in Ghana suggests unidirectional, reverse zoonotic transmission at the human–animal interface	Duplicate
